# West Asian sources of the Eurasian component in Ethiopians: a reassessment

**DOI:** 10.1038/s41598-019-55344-y

**Published:** 2019-12-11

**Authors:** Ludovica Molinaro, Francesco Montinaro, Burak Yelmen, Davide Marnetto, Doron M. Behar, Toomas Kivisild, Luca Pagani

**Affiliations:** 1 Estonian Biocentre, Institute of Genomics, University of Tartu, Tartu, 51010 Estonia; 2 Department of Evolutionary Biology, Institute of Molecular and Cell Biology, Tartu, 51010 Estonia; 3 Genomic Research Center, Gene by Gene, Houston, 77008 Texas USA; 4 Department of Human Genetics, KU Leuven, Leuven, 3000 Belgium; 5 Department of Biology, University of Padova, Padova, 35121 Italy

**Keywords:** Evolution, Genetics

## Abstract

The presence of genomic signatures of Eurasian origin in contemporary Ethiopians has been reported by several authors and estimated to have arrived in the area from 3000 years ago. Several studies reported plausible source populations for such a signature, using haplotype based methods on modern data or single-site methods on modern or ancient data. These studies did not reach a consensus and suggested an Anatolian or Sardinia-like proxy, broadly Levantine or Neolithic Levantine as possible sources. We demonstrate, however, that the deeply divergent, autochthonous African component which accounts for ~50% of most contemporary Ethiopian genomes, affects the overall allele frequency spectrum to an extent that makes it hard to control for it and, at once, to discern between subtly different, yet important, Eurasian sources (such as Anatolian or Levant Neolithic ones). Here we re-assess pattern of allele sharing between the Eurasian component of Ethiopians (here called “NAF” for Non African) and ancient and modern proxies. Our results unveil a genomic legacy that may connect the Eurasian genetic component of contemporary Ethiopians with Sea People and with population movements that affected the Mediterranean area and the Levant after the fall of the Minoan civilization.

## Introduction

Previous genome-scale studies of populations living today in Ethiopia have found evidence of recent gene flow from an Eurasian source, dating to the last 3,000 years^1–4^. Haplotype^1^ and genotype based analyses of modern^2,4^ and ancient data (aDNA)^3,5^ have considered Sardinia-like proxy^2^, broadly Levantine^1,4^ or Neolithic Levantine^3^ populations as a range of possible sources for this gene flow. Given its ancient nature and the extent of population movements and replacements that affected West Asia in the last 3000 years, to clarify the demographic past of contemporary Ethiopians the use of aDNA evidence from West Eurasian specimens seems the best available strategy. We speculate, however, that the deeply divergent, autochthonous African component which accounts for ~50% of most contemporary Ethiopian genomes may affect their overall allele frequency spectrum. This would make it hard to discern between subtly different, yet important, Eurasian sources (such as Anatolian or Levant Neolithic ones). One way to control for this potential confounder is to systematically remove the African component from contemporary Ethiopian genomes through ancestry deconvolution, and to apply the allele sharing tests only on their Eurasian component. Our results demonstrate that such an approach is viable, virtually unbiased and that the results we obtain are qualitatively different and informative on the Iron Age populations that brought a distinctive Eurasian component to North-East Africa.

## Results

**Figure 1:**
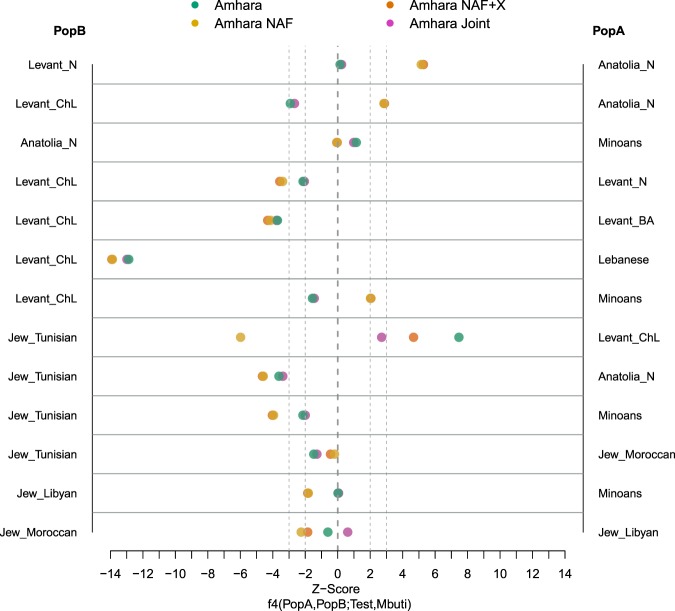
Frequency-based allele-sharing analyses. *f4* statistic test on Amhara in form of (PopA,PopB;Test,Mbuti) to assess genetic similarity between Amhara and respective NAF genomes to pairs of several West Asian populations. A and B populations are listed in the right and left  side of the plot, respectively. Values in x axis indicate the Z-Scores, we draw two lines to highlight |z-Scores| = 2 and 3. Points with |z-Score| > 3 indicate a clear affinity of the test population towards one of the other population. Amhara’s segments tested: Amhara whole-genome (Amhara, in blue), the Non African component (Amhara NAF, in yellow), Amhara AF and NAF components together (Amhara Joint, in violet) and Amhara NAF with X component (Amhara NAF + X, in orange).

To determine the most likely source of the Eurasian gene flow into the ancestral gene pool of present-day Ethiopians we have used a combination of ancestry deconvolution (AD) and allele sharing methods as previously proposed elsewhere^6^. AD refers to analyses that determine the most likely ancestry composition of genomes of individuals with mixed ancestry at fine haplotype resolution. This approach allowed us to (i) exploit high quality modern data and (ii) maximize the power of allele sharing tools on genetic fractions with no or reduced African contributions. Such a strategy, while potentially beneficial, may introduce a novel source of bias which we aimed to explore here. Particularly, after AD of 120 Ethiopian genomes^7^, we assigned each genomic SNP into one of the following four categories based on the method output (see Methods for further details): (1) high confidence non African (NAF); (2) low confidence non African (X); (3) low confidence African (Y) and (4) high confidence African (AF, consistently filtered out from our analyses). While basing our inference on the NAF component alone, we here demonstrate that the component X accounts for just a minority of the genome and, when analysed together with NAF does not qualitatively affect our inferences (Fig. 1 and Supplementary Table S3). Furthermore, we assembled “Joint” components by joining together the high confidence NAF and AF genomic sections of each individual and recapitulated the signals of the global population (prior to ancestry deconvolution). This results shows that the X and Y components are not holding a considerable or peculiar genetic signature. By this way we ruled out, in this study, the role of ancestry deconvolution as a potential source of artifacts. We further explore potential confounders linked with the choice of West Eurasian and African populations as sources, or software^8^ used to perform AD, and confirmed the robustness of our approach through a series of *f4* tests (see Supplementary Fig. S9). For the sake of clarity, out of the four admixed Ethiopian populations available from Pagani *et al*.^7^ (Amhara, Oromo, Ethiopian Somali, Wolayta), we report in the main figures only results on the NAF component of Amhara. Comparable results for the other three populations, which we chose not to lump into a heterogeneous Ethiopian super-population to emphasize potential population-specific peculiarities, are provided in Supplementary Information.

In the following paragraphs we will base our results on the assumption that the majority of the Eurasian component observed in contemporary Ethiopians is the result of a major admixture event that took place ~3 kya. An alternative or complementary contribution to the presence of West Asian components in contemporary Ethiopians, may involve the Neolithic Pastoralist population movements reported to have occurred in East Africa by Prendergast and collaborators^9^. We explored this possibility through MALDER and showed no multiple admixture events in the area (with the exception of Wolayta who show an additional signal for a more recent admixture wave). Even though the events reported by Prendergast and colleagues are at the edge of the MALDER detactability (See Table S2), the lack of admixture dates in Ethiopians prior to 3 kya may point to a reduced impact of this early migrations on Ethiopians, also in accordance with the ancestry modelling suggested for Ethiopian populations by Prendergast and colleagues themselves^9^.

**Figure 2:**
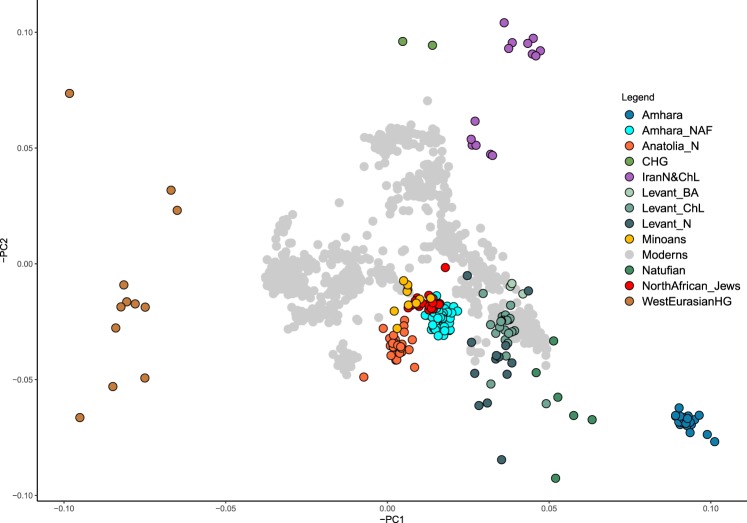
Principal Component Analysis. Principal component analysis of modern West Eurasian populations used as a scaffold (grey points) on which ancient genomes and Amhara deconvoluted NAF were projected. To highlight reference populations we coloured European Hunther-Gatherers in brown, ancient genomes from Anatolia and Levant areas (orange and green respectively), Minoans in yellow, Iranians in purple and Jewish populations from North Africa in red. Amhara whole and NAF genomes are listed in blue and light blue. Variance explained by PC1 is 0.9% and PC2 is 0.3%.

An exploration of the AF component of contemporary Ethiopians (see Supplementary Fig. S6) shows that, prior to the Eurasian arrival in the area, the sampled populations could be described as falling within a relatively homogeneous East African diversity focus, of which contemporary Gumuz seem to be the most plausible representative among available samples. Furthermore our results seem to indicate a reduced or absent genetic impact of the West African Bantu expansion in the area. We then explored the Ethiopian NAF component through ADMIXTURE (Fig. S7) and projected PCA, and showed them to fall within the range of Eurasian populations, close to ancient populations with a high Anatolian Neolithic component (e.g. Anatolia_N and Minoans) and away from neighboring populations from the Arabian Peninsula (Figs. 2 and S2 for Amhara). The PCA position shown by Amhara in Fig. 2 is superimposable to the ones of Oromo, Ethiopian Somali and Wolayta NAF components (Figs. S3–S5) accounting for overall homogeneity of the Ethiopian NAF components extracted by AD. Notably, several Jewish populations from North Africa cluster with NAF as well. The affinity between Anatolian Neolithic and NAF was further highlighted by Outgroup *f3* statistic, in contrast to results obtained with the genomes before ancestry deconvolution (Supplementary Fig. S8). Overall, whole-genome sequences of all the Ethiopian populations appear closer to ancient broadly West Asian populations such as: Minoans, Natufian, Levant Neolithic and Anatolian Neolithic. On the other hand, their NAF components appear closer to populations with a high Anatolian rather than Levantine component (such as Minoans, Sardinians and Anatolia Neolithic). North African (Tunisian, Libyan and Moroccan) Jews (See Fig. S6), consistently with what seen in PCA (See Figs. 2 and S2–S5), show the highest increase of Outgroup *f3* affinity when replacing Ethiopian populations with their NAF counterparts. Importantly, other populations that could have served as good proxy for the Eurasian component in the Ethiopians due their chronological or geographical position (i.e. Sidon_BA^10^, Levant_BA^3^, Iranians^3,11^ (contemporary, Chalcolithic and Neolithic individuals), Egyptians, Yemeni and Saudis^12^), did not show high similarity to the NAF component based on the Outgroup *f3* test and were not further investigated.

**Figure 3:**
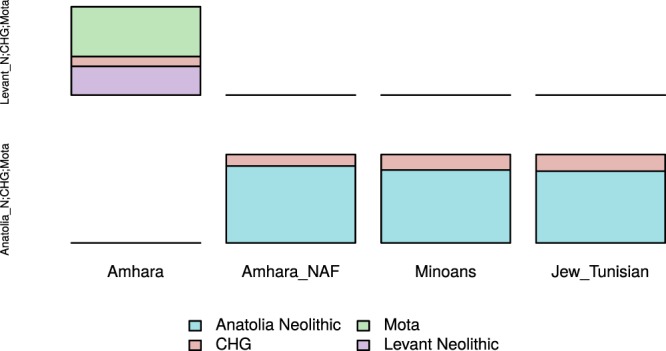
Estimating admixture proportions in the studied populations. Modelling Amhara, Amhara_NAF, Minoans and Jews from Tunisia as a mixture of Mota and West Asian populations, with 2 and 3 way admixtures. Violet indicates the Levantine component, pink the Caucasus Hunter-Gatherers, light green the African component and light blue highlights the Anatolian ancestry. The left side of the graph lists the sources used to model the populations in the x axis; unfilled boxes indicate unfeasible results or p-value < 0.01.

We further dissected the observed affinity between NAF and Anatolian Neolithic-like populations through a set of *f4* tests aimed at refining through more and more stringent comparisons the best proxy population for the Eurasian layer (Fig. 1). The whole-genomes, with both African and Non-African components, are significantly closer to a Levantine ancestry rather than Anatolian (Z-Score −2.98), with them being closer to Levant_ChL individuals than Levant_N. On the other hand, NAF is shown to be closer to a Neolithic ancestry from Anatolia rather than any Levantine one (Z-score 2.847) and, among Levantine populations, notably closer to Levantine Chalcolithic than to Bronze Age groups or contemporary Lebanese. We further compared the best proxies for the Non African component using the top scoring populations from Outgroup *f3* analyses. Minoans appear to be as close to NAF as Anatolian Neolithic individuals (Z-Scores < 1). When we delved into the North African Jews signals, they broadly show affinity with NAF with particular reference to Jews from Tunisian. Similar trends did not change when considering alternative combinations of deconvoluted components, such as NAF + X and Joint (Fig. 1). The similarity between the NAF and Anatolia_N samples, rather than Levantine, is maintained also when different proxy populations are used to extract the NAF component or a different deconvolution software is used (Fig. S9). The *f4* results on the other Ethiopian populations are strongly comparable with Amhara results: Oromo, Ethiopian Somali and Wolayta show higher genetic affinity with Anatolian Neolithic group rather than any Levantine one (Fig. S10), with them being closer to Levantine Chalcolithic individuals rather than Neolithic ones, as seen for Amhara. Peculiarly, Ethiopian Somali and Wolayta when tested specifically with Minoans and Levant Chalcolithic samples show Z-Scores < 2. Given that our ability to pinpoint the actual source of the NAF component is inherently limited by the availability of ancient and modern populations, we used qpGraph (Supplementary Figs. S11–S13) and qpAdm to model NAF as a mixture of the major axes of genetic diversity that best described the Mediterranean area at the time of the studied event, following Lazaridis *et al*.^3^. When looking at the global genomes, for all Ethiopian populations, our qpAdm results replicate a Levant_N origin for the Eurasian component of Ethiopians^3^ (Fig. 3, left column and Fig. S14, first row). The NAF component alone, on the other hand, can be described as a mixture of Anatolia_N and CHG. Particularly, Amhara, Oromo and Wolayta NAF components can be modelled as ~85% Anatolian_N and ~15% CHG, while Ethiopian Somali NAF is better characterized as 92% of Anatolia_N and 8% of CHG. In sum, similarly to Minoan and Tunisian Jewish populations, the non African component of Ethiopians can be best modelled as a mixture of ~85% Anatolian_N and ~15% CHG composition of ancestries (Fig. 3, columns 2, 3, 4 and Table S4), with small fluctuations across the various Ethiopian populations (see Table S4 and Supplementary Fig. S14).

## Discussion

Our analyses aimed at describing the non African component of Ethiopians as a combination of available ancient ones, and we stress our results should not be interpreted as involving a direct connection or descent line between Neolithic Anatolia and Ethiopia. Instead, these results can be seen as informative for shortlisting available ancient and modern populations which, following geographic and chronological considerations, may be suitable proxies for the groups that mediated the Eurasian gene flow to East Africa. Of the ones analyzed here, Minoans and Tunisian Jews seem to provide the two closest matches to NAF, adding on top of the genetic evidence a criteria of space/time compatibility. A tentative link between these three groups may be provided by the historical maritime trade routes connecting Crete (home to the Minoan culture) to the Levant^13–15^ and by the shuffling role played by a horde of nomads who navigated throughout the Mediterranean Sea 3 kya: the Sea People. These tribes are linked to Crete, Anatolia where they fought the Hittite Empire, Egypt and the Levant, and are told to have settled in the land of Canaan, known also as Palestine^16^. Interestingly, the Sea People tribes that settled in Palestine included, among others, Denyen and Peleset according to the Egyptian inscriptions of Merneptah and Medinet Habu^17^. Although there are different theories around the origin of each of these tribes, there are suggestions that link the Denyen with the tribe of Dan, from which Jews from Ethiopia have been said to descend, and the Peleset to the Philistines from the Levant^18^. The role of Sea People may therefore be crucial in explaining a temporary presence of a Minoan-like ancestry in the Levant, bringing Anatolian-like components to levels as high as 85%. A pulse of populations with Anatolian-rich ancestry has just been recently detected in Iron Age Levant, appearing and disappearing from the archaeological record within a range of few centuries, at the beginning of the 1st Millennium BCE^19^. These Levant Iron Age samples can indeed be modelled as having at least 80% Anatolian Neolithic ancestry (~20% CHG and ~80% Anatolia_N, see Supplementary Table S6) and surrogate Ethiopian NAF in relevant *f4* analyses (Supplementary Table S5). Notably Ethiopian NAF is still closer than Levant IA to Tunisian Jews (Supplementary Table S5). Ethiopian NAF therefore offers a solution to the disappearance of the Levant IA component from the population record of the area, where their signal may have become erased as a consequence of major warfare after 1000 BCE^20^ or 3 kya, displacing these genetic components towards Ethiopia (an allegory of which can be read in the mythological account of the meeting between King Solomon and the Queen of Sheba) and North Africa Jewish communities (where such a signature is still detectable after the major population movements following the Alhambra Decree after 1492 CE).

In conclusion, our work shows that when the mixing components are deeply differentiated, such as in the case of contemporary Ethiopians, ancestry deconvolution increases the sensitivity of allele sharing tests and enables to fully exploit the high quality of modern genomes.

## Methods

### Dataset and Samples

We merged different available datasets containing both ancient and modern DNA, African and Eurasian populations from the following publications^1,3,5,7,10–12,19,21–37^. Northeast African populations whole-genome sequences were taken from Pagani *et al*.^7^, and included 5 modern Ethiopian populations: Amhara, Gumuz, Oromo, Ethiopian Somali and Wolayta. We chose to focus on the whole genome sequence data rather than on SNP arrays^1^ to increase the number of available SNPs to be compared with aDNA and other references. To maximize the number of individuals typed at each SNP, we downsampled the dataset to 1037084 markers to match the ones of the extended Human Origin Array on which most of the ancient DNA samples were typed. For ease of exposition we chose Amhara, the population with the highest Eurasian fraction among the available ones^7^, to represent all main results. We provide full description of all other Ethiopian populations in Supplementary Material, except for Gumuz, who have negligible Eurasian traces^7^, and whose NAF component was not studied. Similarly, we chose not to group all the available samples within a single “Ethiopian” population, to allow for group-specific stories to emerge.

We plotted the study and reference populations on a geographical map (See Supplementary Fig. S1) with R using ggmap package^38^.

### Ancestry Deconvolution and dating

#### Subsetting Modern Genomes

We started with dating the mixture time and number of waves of admixture using MALDER^2^ with mindis parameter set as 0.005. We perfomed MALDER on whole-genome sequences from Pagani *et al*.^7^ and on SNP Array data from Pagani *et al*.^1^. To evaluate possible differences between whole-genome and SNP Array results, that could arise due to the different number of SNPs, we downsampled the whole-genome dataset to match SNP Array markers and performed MALDER on the resulting subsetted dataset. From phased genomes, we refined the ancestral components identification in Eastern Africans individuals provided by Pagani *et al*.^7^ with PCAdmix^39^. For every 20 SNPs window of the genome, there is a probability to have a source of African (AF) ancestry or Non African (NAF) ancestry (in which case the probability is 1 – AF), which is given by fbk values and refined with Viterbi algorithm^40^. We set a fbk threshold of 0.9 probability in order to assign every window to either one layer of ancestry or the other. Windows not reaching the threshold for any component, were labeled as unassigned. CEU (Utah residents with ancestry from northern and western Europe) were used as a proxy for the Non African component, and Gumuz (the Ethiopian population showing minimal Eurasian component) were used as a proxy for the African component following Pagani *et al*.^7^. Once the ancestral components were detected, we created the “Genomes Subsets” using the windows that reached the specified threshold. The “Genomes Subsets” are genomes in which for every haplotype only the high confidence African or Non African component is retained, while the rest is assigned as “missing data”. Therefore, “Genomes Subsets” are partial genomes in which only the haplotypes derived from a specific ancestry (either African or Non African) are present (see Yelmen *et al*.^6^ for further details). The ancestry deconvolution process has been applied to East African populations only from Pagani *et al*.^7^ populations, namely: Amhara, Gumuz, Oromo, Ethiopian Somali and Wolayta.

#### Sifting through all possible ancestry fractions

To test for possible biases introduced by using CEU as proxy for the Non African component, we further divided the deconvolution results into different segments to investigate specifically the parts of the genome that were not assigned to either ancestry. We retrieved the different components from the fbk values alone, without refining them with the Viterbi algorithm, to maintain all possible segments information. For each of the two ancestries we obtained two components: X and Y, which held the sequences assigned with 51–90% and 10–50% respectively, representing the unassigned sequences in the masking process. The component X is made of sequences that were not assigned to NAF, representing the unassigned segments that we expect to bear Eurasian traces along with spurious African ones; the component Y is made of segments which we expect to be characterized mainly by African traces. The X and Y segments correspond each to 7% of the genome, and we expect their contribution to the final results to be minimal.

### Principal Component and ADMIXTURE Analyses

We performed PCA as an initial screening method on the dataset with smartpca from EIGENSOFT^41,42^, using the lsqproject option and autoshrink:YES. For the analyses focused on the NAF component we used modern European and West Asian populations with minimal missingness (–geno 0.1 with PLINK^43^) to compute PCs and projected the rest of the samples included the ancient samples and the Ethiopian NAF genomes. For the analyses focused on the AF component we used modern African populations as scaffold, while ancient and AF individuals were projected. We used ADMIXTURE^44^ software to perform supervised clustering of ancient and decolvoluted NAF genomes using as reference modern European and West Asian genomes along with Yoruba as West African, Gumuz as East African and Han as East Asian. We used R and ggplot2 package for visualization^45,46^.

### Frequency-Based Allele-Sharing Analyses

We used POPSTATS^47^ to calculate Outgroup *f3* statistic in the form of *f3*(Test, A, Mbuti) with Test being the Ethiopian whole-genome sequences and the NAF individuals, and A being the set of all possible chronological and geographical proxies for the admixture (See Supplmentary Table S1). To further infer the Non African component we used Admixtools 4.1^30^. We performed *f4* analyses using qpDstat along with the option f4mode:YES with this format: A,B;Test,O. As Test populations we used both whole-genome sequences and NAF components of Amhara, Ethiopian Somali, Wolayta and Oromo. As A and B we used pairs of top scoring populations obtained from Outgroup *f3* analyses. With Admixtools we performed qpWave and qpAdm with the set of Right populations firstly defined by Lazaridis^3^, with the exception of Onge, which is not present in our analyses. Right populations used: Ust_Ishim, Kostenki14, MA1, Han, Papuan, Chukchi, Karitiana, EHG, Natufian, Switzerland_HG, WHG. We reported qpAdm results that show significance <0.001 in qpWave, which was performed with the set of Left populations, without the Test population. We used for every analysis a custom list of Left populations to test a two-way or a three-way admixture. The Left populations used to perform qpAdm were selected in this order: the Test population, A and Mota for the two-way admixture; the Test population, A, CHG and Mota for the three-way admixture. Here A stands for either Levant_N or Anatolian_N groups. We reported both significant and non significant results as they both might be indicative for the purpose of our analyses. We set our threshold to accept a result as significant at 0.01. We then used the information gathered from qpAdm to build a qpGraph model. We proceeded modelling qpGraph tree starting from a simple tree topology, then adding populations of interest at each step and modifying the topology to minimize the *f2* and *f4* Z-Score values.

### Bias Testing

We performed further analyses on Amhara, our representative population, in order to detect in the unassigned sequences (X and Y components) whether important signal were lost in the deconvolution process. We compared our test populations with the *f4* statistic using this format: A,B,Test,O. As Test populations we used: Ethiopians whole genome sequences, NAF genomes, Ethiopians_J, where “J” stands for “Joint”. The Joint individuals, created for each population with sizeable Eurasian contribution (Amhara, Oromo, Ethiopian Somali and Wolayta), are built as a synthetic population made of the NAF and AF sequences refined by the Viterbi algorithm that passed the fbk 90% threshold, and thus not yielding the unassigned segments. We then added the X segments to NAF to test whether the unassigned component would give different results from the Non-African component NAF alone, which would indicate presence of biases in the deconvolution step. As A and B we used the possible proxy populations that may have contributed to the admixture: Levant_N, Anatolia_N, Levant_ChL. We modelled the NAF along the X component with qpAdm, using the same Left and Right populations used for the main analyses to investigate how the X component can be modelled and if the NAF with the addition of X could be modelled as the Non African component, which could indicate no bias.

We also explored the impact on our results of the proxy populations chosen for the ancestry deconvolution step and of the deconvolution software itself. Starting from our initial Gumuz-CEU pair of proxies, we used PCAdmix as described to deconvolute Ethiopians using CEU-YRI and Gumuz-Druze as alternative pairs of proxies. On one hand, YRI (Yoruba from Ibadan, Nigeria), a Western African population, was chosen to maximize the distance of the African proxy to the population that was likely involved in the admixture event. On the other hand Druze, a Levantine population showing little or no sign of recent African admixture^48^, was chosen to minimize the distance from the true West Eurasian source. We then applied the same set of *f4* tests using these newly obtained NAF components and showed no qualitative difference from our previous results. Once shown the reliability of Gumuz-CEU as a proxy pair, we also performed the deconvolution with ELAI^8^, a novel software shown to outperform several other deconvolution approaches. We run ELAI 10 times and averaged the results, setting 100 generations as admixture time, based on Pagani and collaborators^1,7^, and 20 EM steps according to the manual. We retained the genomic regions assigned to NAF and, also in this case, replicated our *f4* results showing that neither the choice of population proxies nor the deconvolution software are introducing perturbations capable of qualitatively altering our conclusions.

## Supplementary information

Supplementary Information

Supplementary Information

Supplementary Information

Supplementary Information

Supplementary Information

Supplementary Information

Supplementary Information
