# Supplementary material for: West Asian sources of the Eurasian component in Ethiopians: a reassessment
Source: Sci Rep. 2019 Dec 11;9:18811. doi: 10.1038/s41598-019-55344-y (PMC6906521; doi:10.1038/s41598-019-55344-y)
Supplement: Supplementary file 1 — Supplementary Information [file 41598_2019_55344_MOESM1_ESM.pdf]

West Asian sources of the Eurasian component in Ethiopians: a reassessment

Ludovica Molinaro[1,2*], Francesco Montinaro[1], Burak Yelmen[1,2], Davide Marnetto[1], Doron M. Behar[1,3], Toomas Kivisild[1,4], Luca Pagani[1,5]

[1]Estonian Biocentre, Institute of Genomics, University of Tartu, 51010, Estonia
[2]Department of Evolutionary Biology, Institute of Molecular and Cell Biology, 51010, Estonia
[3]Genomic Research Center, Gene by Gene, Houston, 77008, Texas, USA
[4]Department of Human Genetics, KU Leuven, 3000, Belgium
[5]Department of Biology, University of Padova, 35121, Italy

*To whom correspondence may be addressed: lu.molinaro8@gmail.com

Supplementary Material

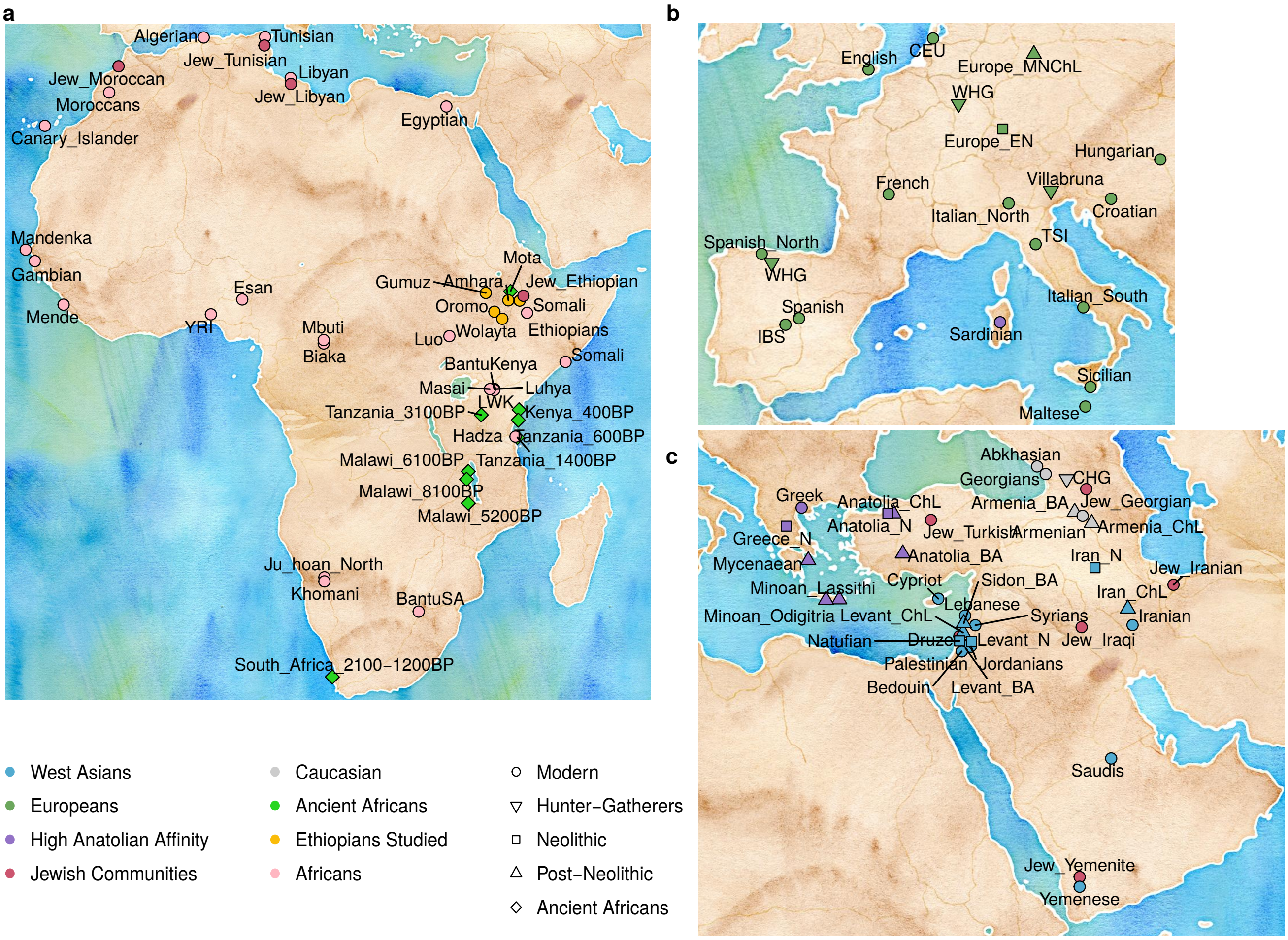

Figure S1: Map of the target and reference populations. Generally, we indicated in purple groups with a high affinity to Anatolian populations and in dark red Jewish groups. In panel a) we listed African populations in pink and ancient African individuals in light green. The Ethiopian samples studied are listed in yellow. In panel b) we listed European populations in dark green. Panel c) lists Middle East, Caucasus and Anatolian populations. In brown we indicated Levantine populations, in grey Caucasus samples. Circles indicate modern samples, flipped triangles Hunter-Gatherers, squares Neolithic individuals, triangles Chalcolithic and Bronze age individuals and rhombus indicates ancient individuals from Africa. (Map tiles by Stamen Design, under CC BY 3.0. Data by OpenStreetMap, under CC BY SA.)

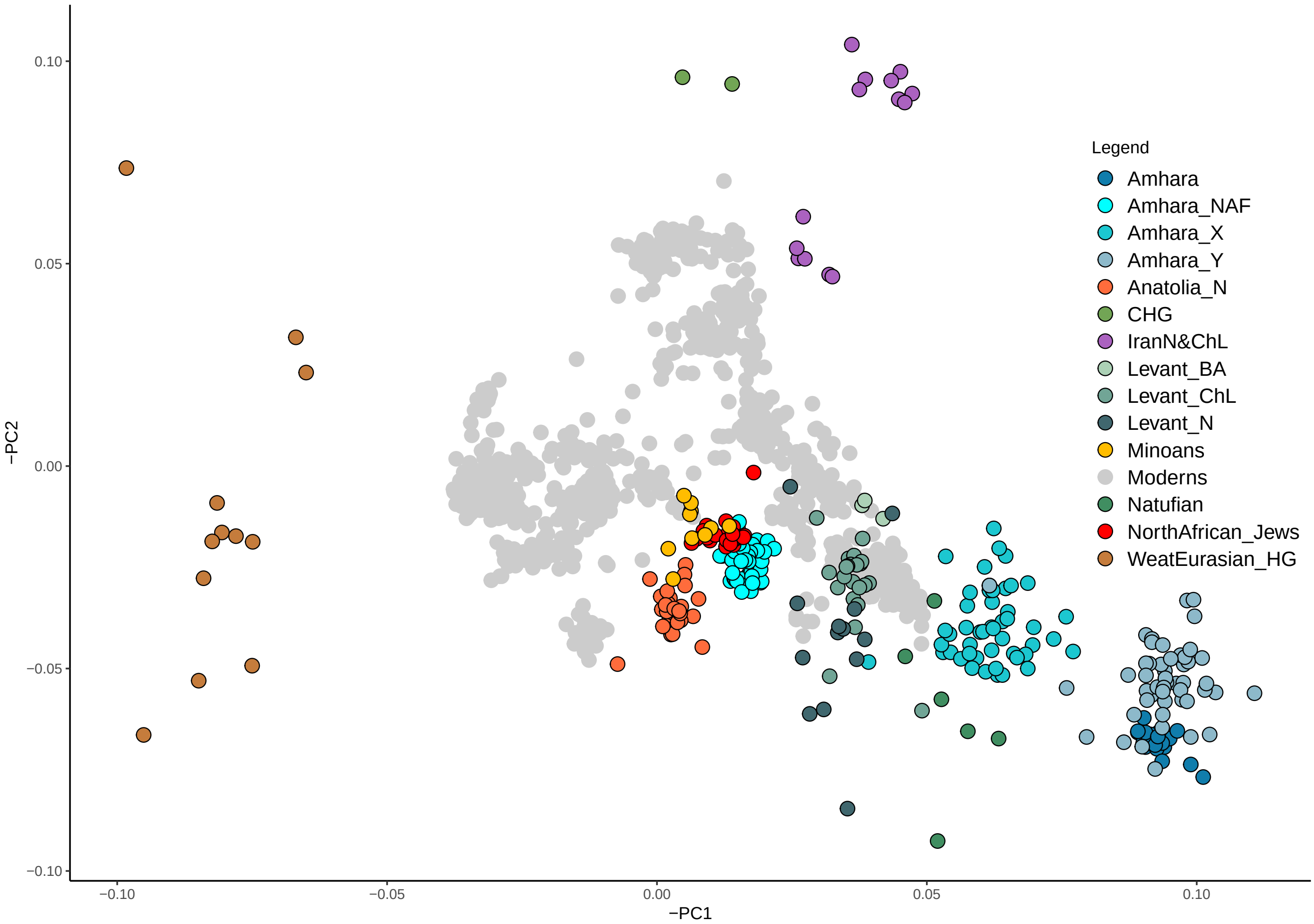

Figure S2: Principal component analysis shown in Figure 2 with the addition of the components extracted to test for biases, specifically for Amhara: X and Y (Amhara_X, Amhara_Y). The X component falls between Levantine samples and Ethiopian whole-genome individuals, as expected due to the unassigned Non African sequences and the spurious African ones. Given that the Y component bears more African traces than the X component, it falls farther right in the PC1, clustering with Amhara whole-genomes. Variance explained by PC1 is 0.9% and PC2 is 0.3%

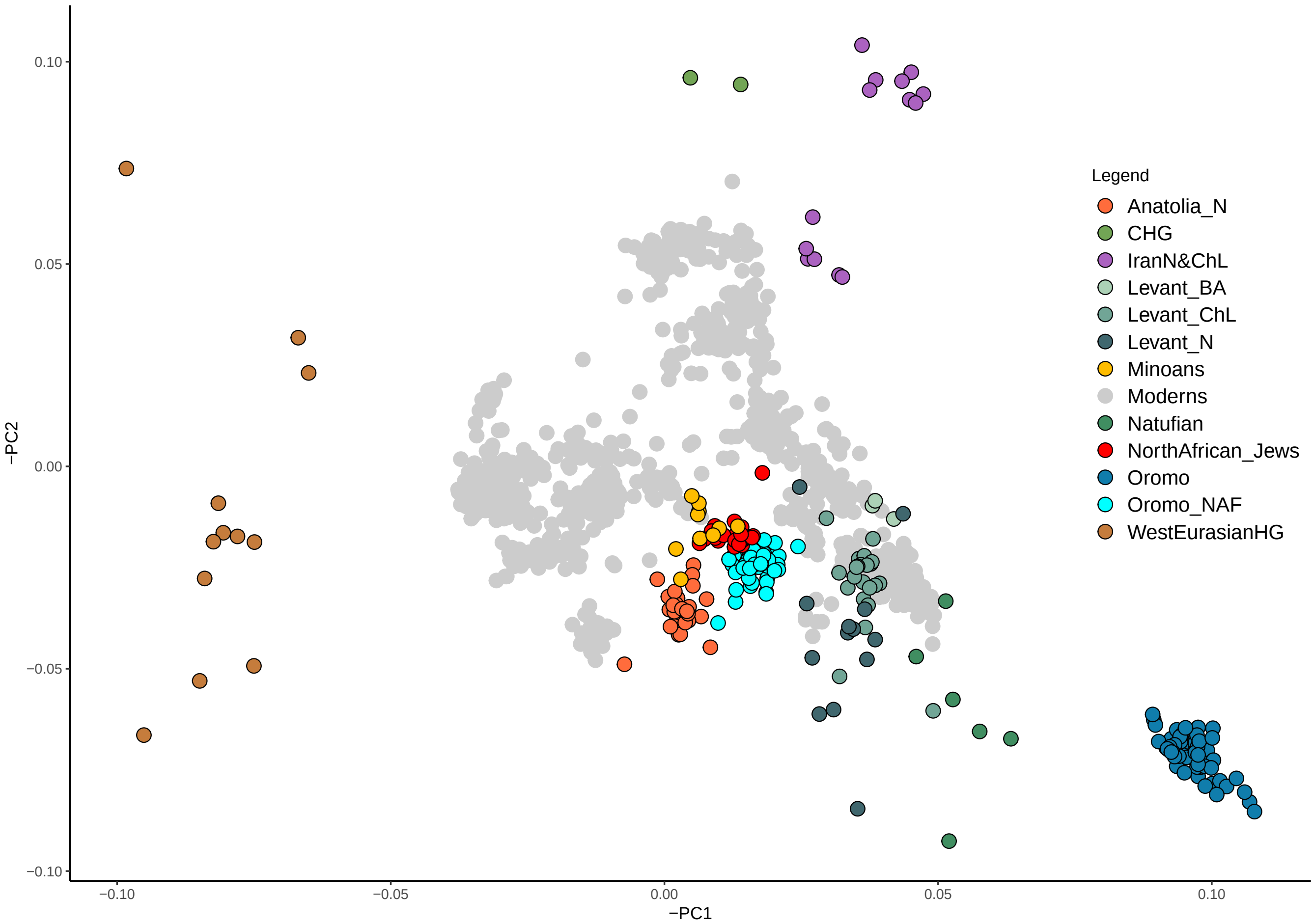

Figure S3: Principal component analysis shown in Figure 2 with only Oromo population indicated, whole-genome in dark blue and NAF sequences in light blue.Variance explained by PC1 is 0.9% and PC2 is 0.3%

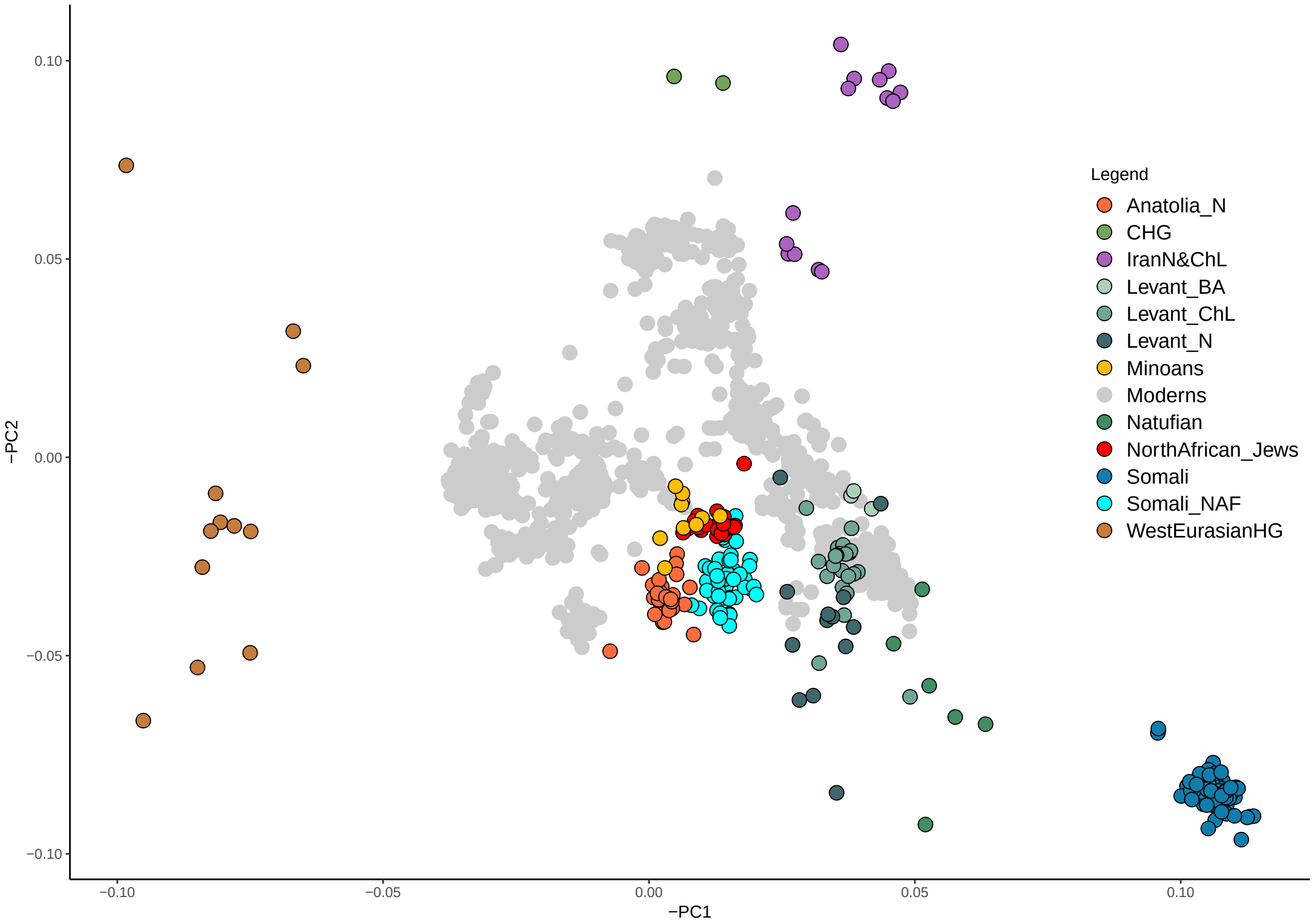

Figure S4: Principal component analysis shown in Figure 2 with only Ethiopian Somali population indicated, whole-genome in dark blue and NAF sequences in light blue.Variance explained by PC1 is 0.9% and PC2 is 0.3%

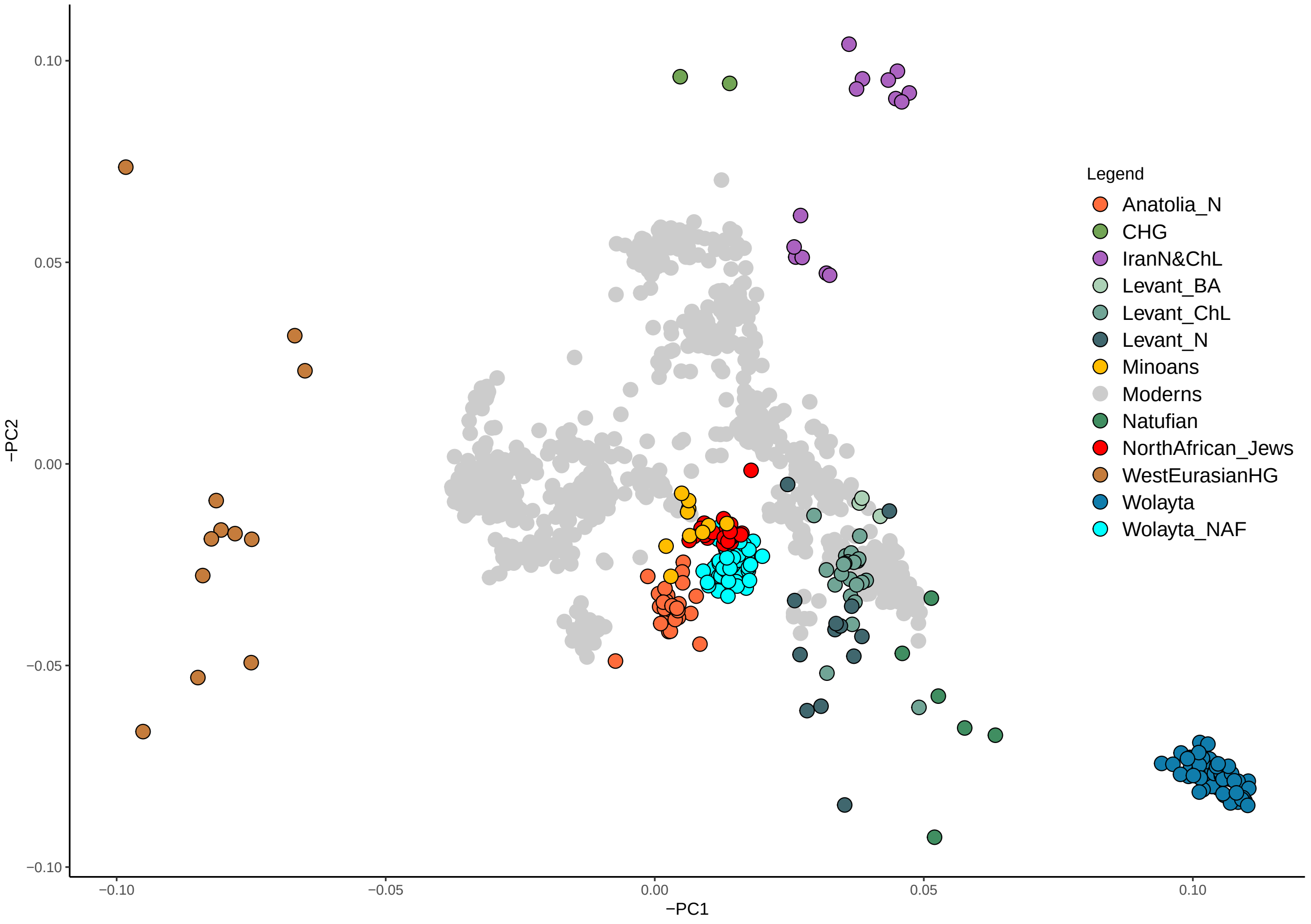

Figure S5: Principal component analysis shown in Figure 2 with only Wolayta population indicated, whole-genome in dark blue and NAF sequences in light blue.Variance explained by PC1 is 0.9% and PC2 is 0.3%

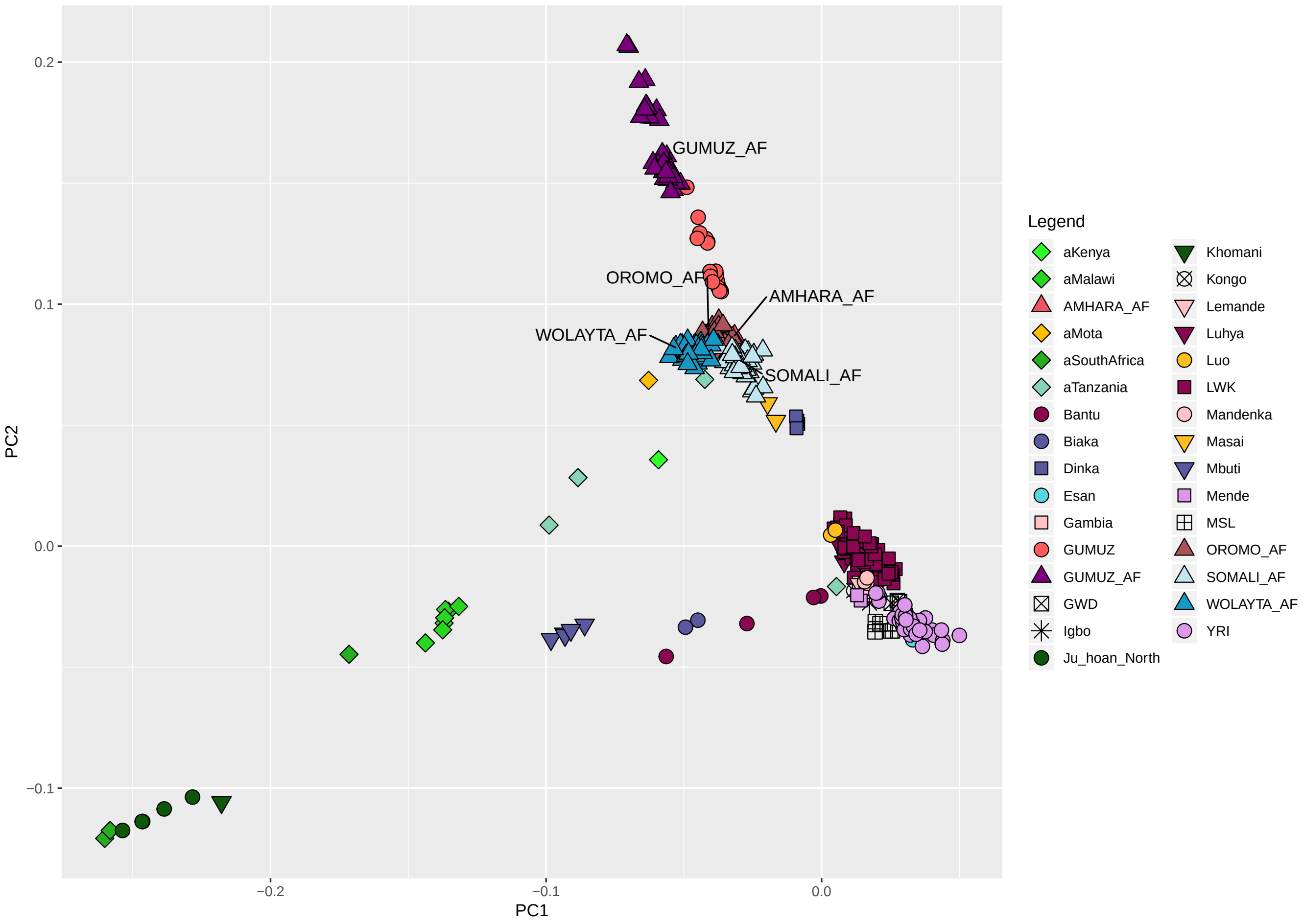

Figure S6: Principal component analysis of Ethiopian masked AF samples (triangles), ancient African individuals (diamond) and modern populations (other shapes).

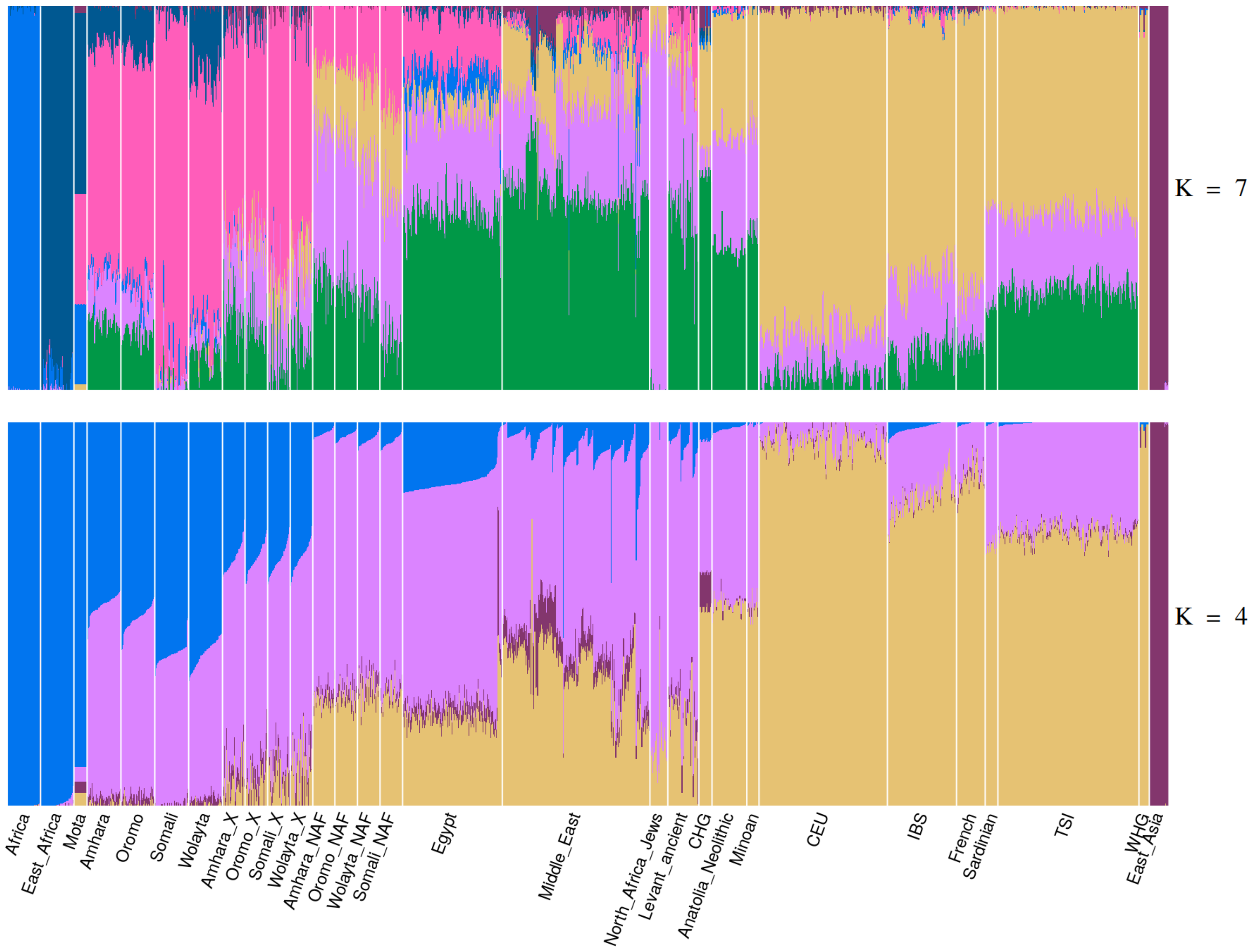

Figure S7: Supervised ADMIXTURE using modern populations as a reference on which we projected ancient and deconvoluted genomes. K=7 shows the smallest cross validation error.

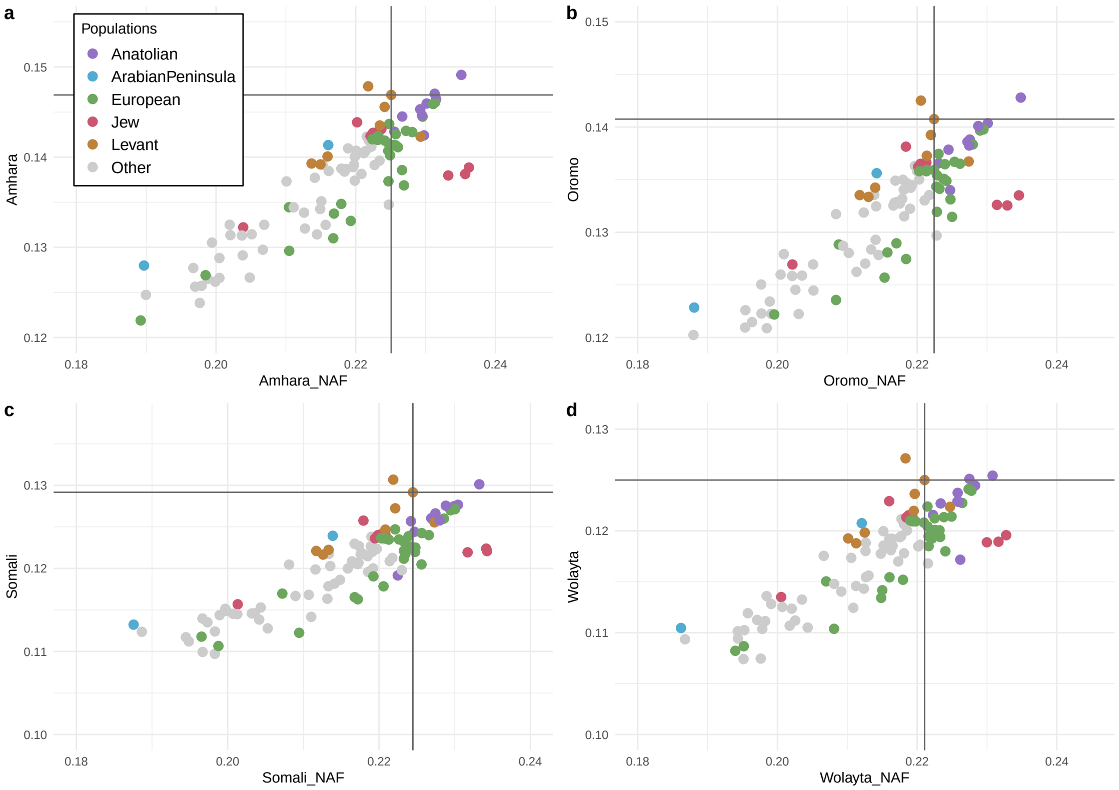

Figure S8: Scatterplot of outgroup *f3* results in form (NAF, X; Mbuti) on the x axis; *f3* in form (Amhara/Oromo/Somali/Wolayta, X; Mbuti) on the y axis, where X stands for several possible genetic and geographic neighbours. Populations are listed and colored based on their geographical origin. The vertical and horizontal lines intersect on Levant_N to highlight the most up-to-date hypothesis of the origin of the Non African component.

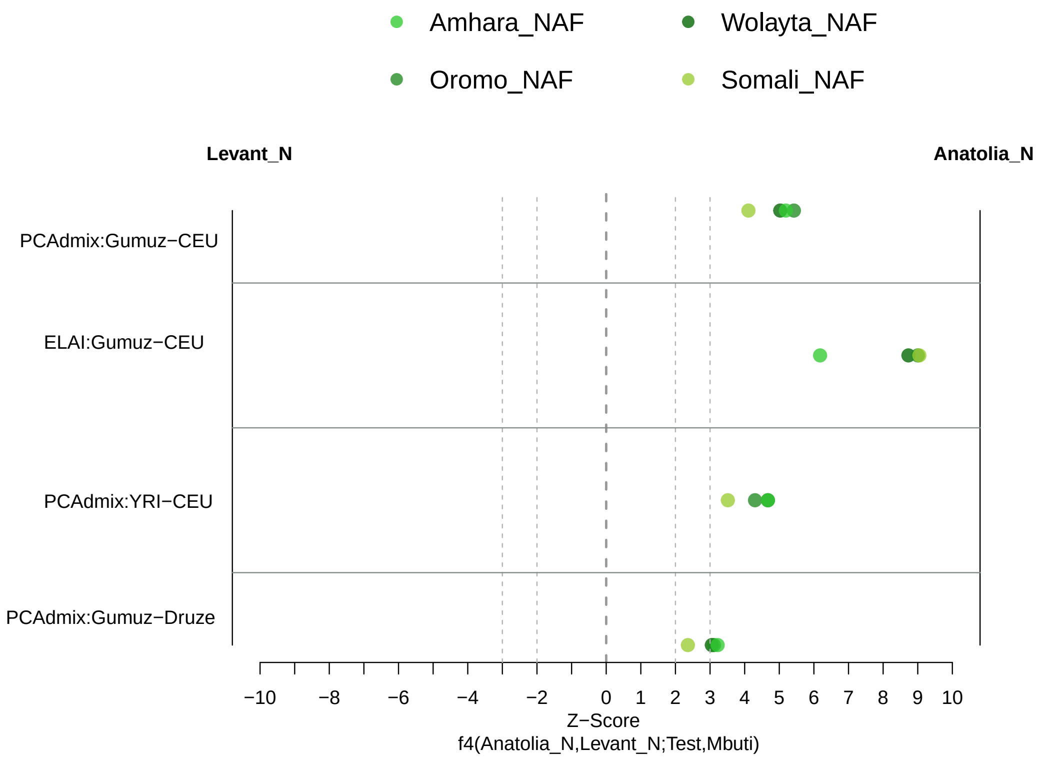

Figure S9: *f4* statistic results on Masked Ethiopians, using either different populations as sources (first,third and fourth rows) or methods to perform Local Ancestry (ELAI:Gumuz-CEU). *f4* on the NAF component were performed in form of (PopA,PopB;Test,Mbuti) to test genetic similarity of the Ethiopians, Levant Neolithic (PopA) and Anatolia Neolithic (PopB). Different methodologies used to retrieve the ancestries are listed in the right and left part of the plot. Values in x axis indicate the Z-Scores, we draw two lines to highlight $|$z-Scores$| = 2$ and 3. Points with $|$z-Score$| > 3$ indicate a clear affinity of the test population towards one of the other population.

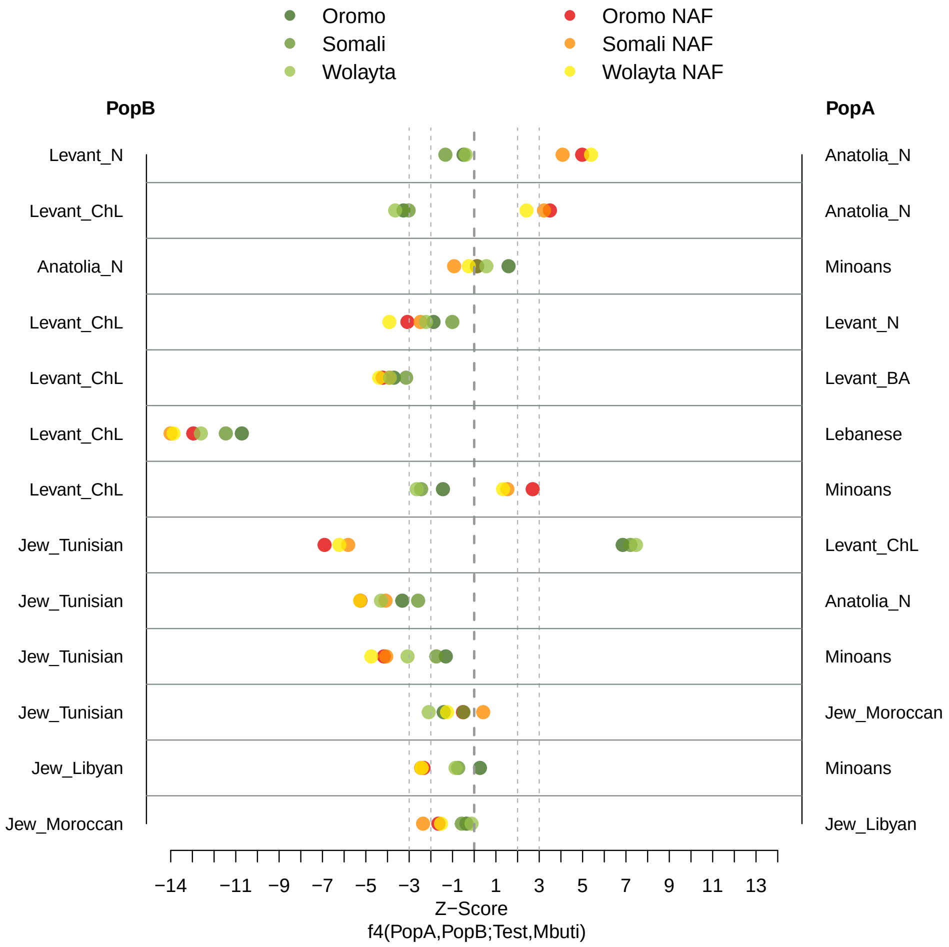

Figure S10: *f4* statistic results on Oromo, Somali and Wolayta in form of (PopA,PopB;Test,Mbuti) to test genetic similarity of the Ethiopians and respective NAF genomes to pairs of Anatolian, Levantine and North African populations. A and B populations are listed in the left and right side of the plot, respectively. Values in x axis indicate the Z-Scores, we draw two lines to highlight |z-Scores|= 2 and 3. Points with |z-Score| > 3 indicate a clear affinity of the test population towards one of the other population.

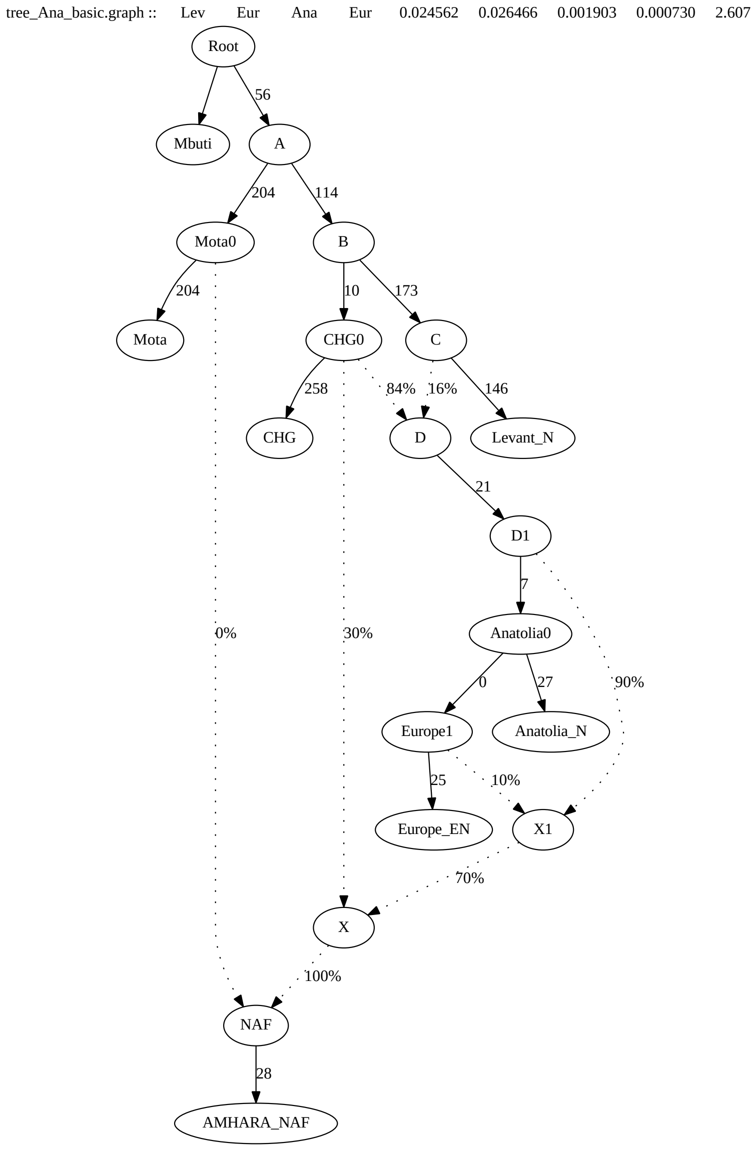

Figure S11: Admixture graph modelling Amhara NAF as being admixed with an African and two Eurasian ancestries. Worst $f4$ = 2.607, dof = 2 and final score = 2149

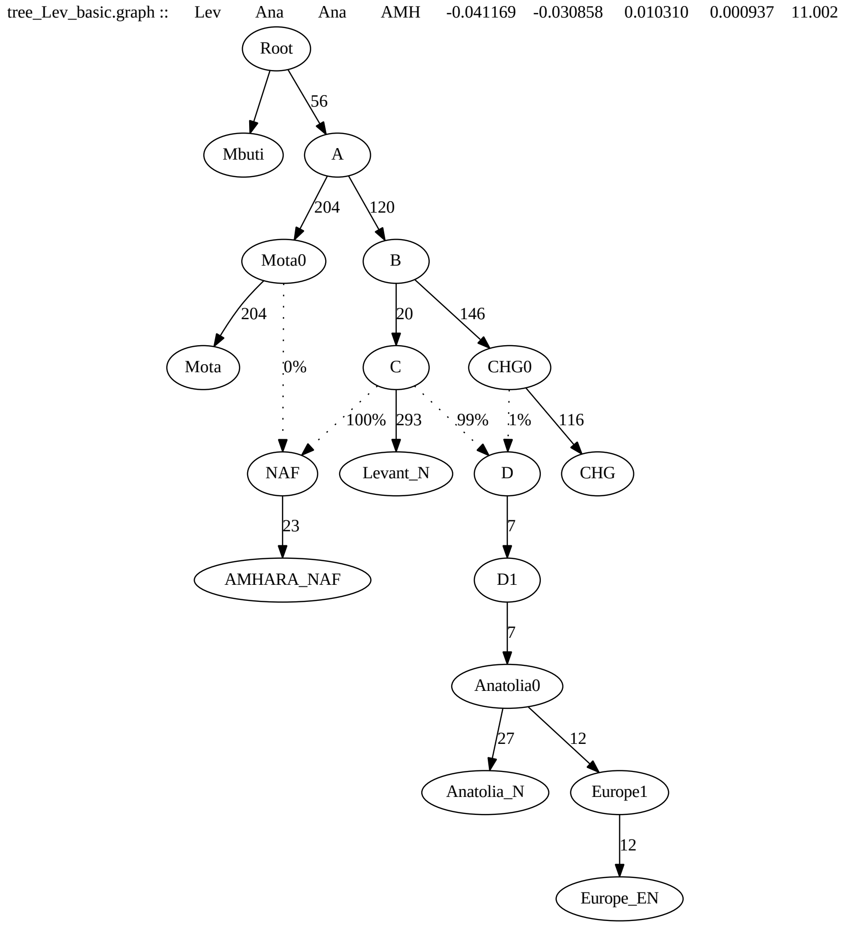

Figure S12: Admixture graph failing to model Amhara NAF as being admixed with an African and two Eurasian ancestries, one of which is Levant_N. Worst *f4* = 11.002, dof=4 and final score = 16522

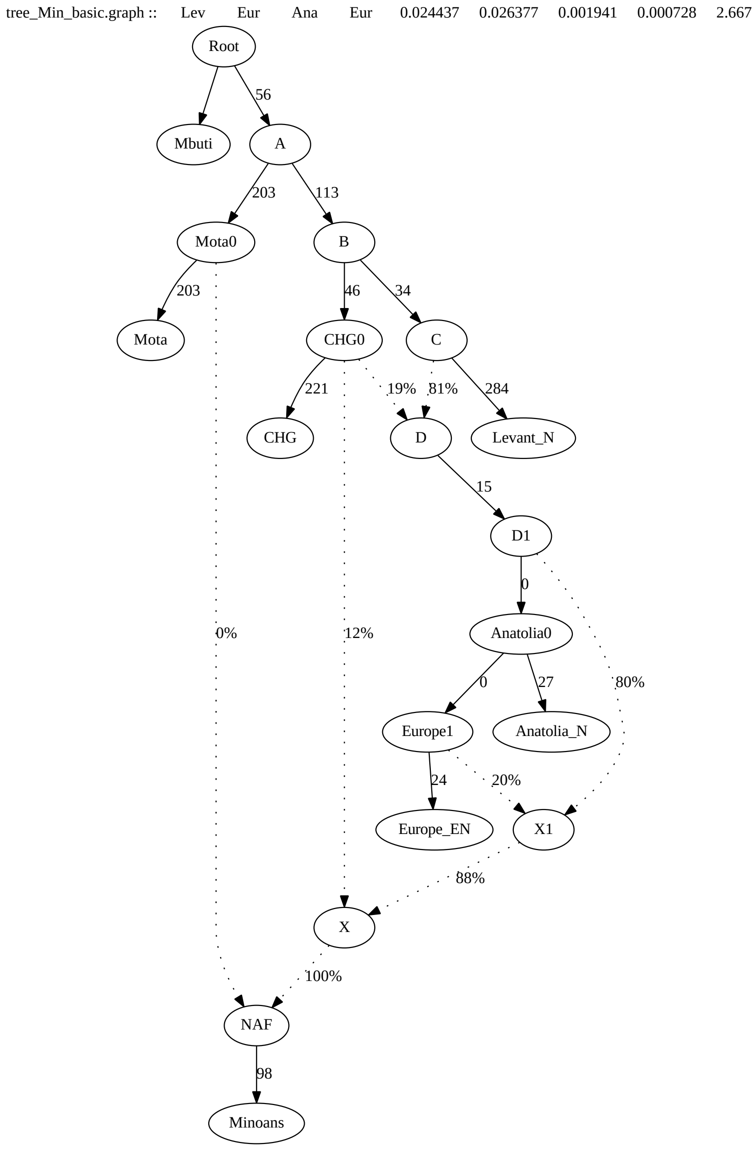

Figure S13: Admixture graph modelling Minoans as being admixed with an African and two Eurasian ancestries. Worst $f4 = 2.667$, dof = 2 and final score = 2046.

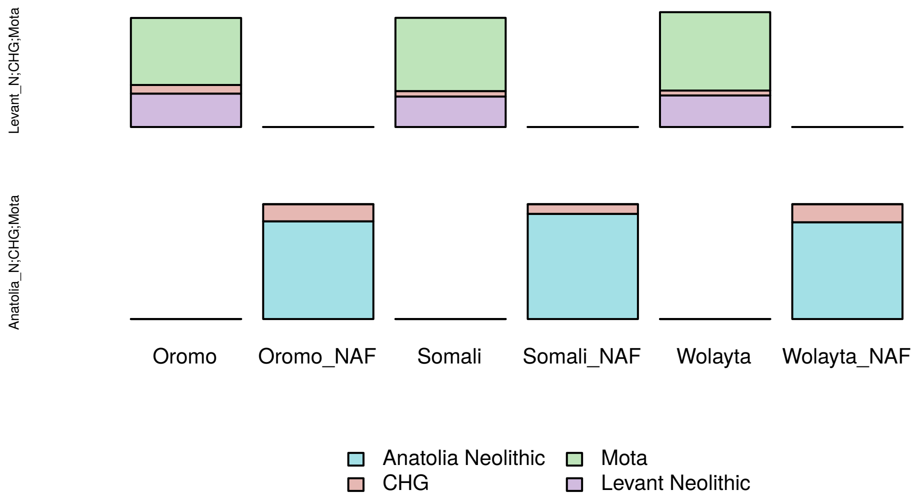

Figure S14: Modelling Oromo, Somali, Wolayta and their respective NAF component as a mix of Mota and Eurasian populations, with 2 and 3 way admixtures. Violet indicates the Levant_N component, pink the Caucasus Hunter-Gatherers, light green the African component and light blue highlights the Anatolian ancestry. The left side of the graph lists the sources used to model the populations in the x axis; unfilled boxes indicate unfeasible results or p-value < 0.01.

Table S1: List of target and reference populations studied. See Supplementary Table S1 provided as a separate file.

Table S2: Inferring admixture dates with MALDER in Amhara, Oromo, Ethiopian Somali and Wolayta whole-genome sequences, snp-chip data and whole-genome SNPs subsetted to match snp-chip data. See Supplementary Table S2 provided as a separate file.

Table S3: *f4* results in form (PopA,PopB; Test,O) testing genetic similarity between Ethiopians (Test) and different Eurasian populations (PopA and PopB). See Supplementary Table S3 provided as a separate file.

Table S4: Modelling Ethiopians and North African Jews as a mixture of Mota and Eurasian populations. P-values > 0.05 are indicated in bold. See Supplementary Table S4 provided as a separate file.

Table S5: *f4* results in form (Ashkelon, AMHARA_NAF; Test,O) testing genetic similarity between different Iron Age Levantine populations and Amhara Eurasian component. See Supplementary Table S5 provided as a separate file.

Table S6: Modelling Ashkelon groups as a mixture of CHG and Anatolia_N. See Supplementary Table S6 provided as a separate file.
